# Ecological and Behavioral Drivers of Supplemental Feeding Use by Roe Deer *Capreolus capreolus* in a Peri-Urban Context

**DOI:** 10.3390/ani10112088

**Published:** 2020-11-10

**Authors:** Federico Ossi, Nathan Ranc, Paul Moorcroft, Priscilla Bonanni, Francesca Cagnacci

**Affiliations:** 1 Centro Agricoltura Alimenti Ambiente, Università degli Studi di Trento, Via Edmund Mach 1, 38010 San Michele all’Adige, Italy; 2 Department of Biodiversity and Molecular Ecology, Research and Innovation Centre, Fondazione Edmund Mach, Via E. Mach 1, 38010 San Michele all’Adige, Italy; nathan.ranc@gmail.com (N.R.); priscilla.bonanni@gmail.com (P.B.); francesca.cagnacci@fmach.it (F.C.); 3 Department of Organismic and Evolutionary Biology, Harvard University, 26 Oxford Street, Cambridge, MA 02138, USA; paul_moorcroft@harvard.edu; 4 Department of Animal and Human Biology, University of Rome “La Sapienza”, Viale dell’Università 32, 00185 Rome, Italy

**Keywords:** camera trapping, competition, diel cycle, generalized additive mixed models, ungulates, urban ecology, wildlife management

## Abstract

**Simple Summary:**

Supplementary feeding of ungulates is widespread in many European countries. However, little is known on the actual patterns of use of feeding sites by animals, and the effects of this practice on inter-individual relationships. We monitored the use of feeding sites in a population of roe deer living in the Italian Alps using camera traps, to enable continuous monitoring of feeding sites and to detect co-occurring visits by multiple individuals. We found that roe deer visits to feeding sites decreased from winter to spring at the onset of vegetation regrowth and when the temperature increases. During the 24 h cycle, roe deer visited feeding sites mainly at crepuscular hours, both solitarily (the majority of the cases) and in groups, with no substantial differences between sexes. When roe deer gathered at feeding sites, they spent more time there, therefore, exhibiting a certain tolerance to the presence of conspecifics. The presence and use of feeding sites did not alter the daily activity cycle and opportunistic use of resources in roe deer, however, they conditioned the intra-specific interactions, with potential behavioral and epidemiological consequences.

**Abstract:**

Winter supplemental feeding of ungulates potentially alters their use of resources and ecological interactions, yet relatively little is known about the patterns of feeding sites use by target populations. We used camera traps to continuously monitor winter and spring feeding site use in a roe deer population living in a peri-urban area in Northern Italy. We combined circular statistics with generalized additive and linear mixed models to analyze the diel and seasonal pattern of roe deer visits to feeding sites, and the behavioral drivers influencing visit duration. Roe deer visits peaked at dawn and dusk, and decreased from winter to spring when vegetation regrows and temperature increases. Roe deer mostly visited feeding sites solitarily; when this was not the case, they stayed longer at the site, especially when conspecifics were eating, but maintained a bimodal diel pattern of visits. These results support an opportunistic use of feeding sites, following seasonal cycles and the roe deer circadian clock. Yet, the attractiveness of these artificial resources has the potential to alter intra-specific relationships, as competition for their use induces gatherings and may extend the contact time between individuals, with potential behavioral and epidemiological consequences.

## 1. Introduction

Winter supplemental feeding of ungulates is a widespread practice in human-dominated landscapes and may occur in both urban and peri-urban (sensu [1]) areas. While this anthropogenic provisioning of food is mainly directed towards ungulates [2], non-target species typically also rely on such resources [3,4]. Supplemental feeding is conducted for various management goals, especially to improve overwinter survival, males’ trophy quality, and more generally individual phenotypic conditions [2]. The provisioning of concentrated artificial food at feeding sites is also often intended to limit field and forest damage [2,5]. While the fulfillment of these objectives is questionable [6,7], the evidence of the drawbacks of such practices is increasing [8,9]. First, excessive consumption of ad-libitum provided food can unbalance the winter diet [10], affecting the microbiota composition and therefore individual health, as observed for roe deer (*Capreolus capreolus*) [11]. Second, supplemental feeding influences spatial behavior by attracting individuals towards these food resources [12], with observed consequences at multiple spatio-temporal scales (alteration of seasonal migration patterns [13]; shrinkage of individual weekly home ranges [14]; alteration of movement and space-use patterns of resident animals [15,16]). In addition, such attraction towards feeding sites can lead to an aggregation of individuals in the proximity of these resources [5,17], especially when population densities are high. Under these circumstances, intraspecific competition for access to food [18,19] and aggressive interactions [20] can be observed. Ultimately, the observed aggregation and competition can significantly raise the risk of disease transmission [21,22], by increasing: (i) the probability and duration of contacts among individuals when considering directly transmitted diseases [23] and (ii) the stress-mediated glucocorticoid concentrations, which can potentially lower immune defenses (see [24] for an example on wapiti (*Cervus canadensis*)).

In this context, understanding the determinants of use and inter-individual behavior of target species in the proximity of feeding sites in urban and peri-urban contexts is crucial to assess the potential side effects of this practice, including disease transmission [17,25]. However, empirical evidence linking the use of feeding sites to underlying environmental and behavioral drivers are surprisingly limited. To this end, it is essential to focus on the effects of these concentrated anthropogenic resources (i.e., specific feeding sites) on individual behavior. This can be solely achieved through continuous monitoring of their use, as allowed by camera traps that record undisturbed individual behavior and intra and/or interspecific relationships [17,26].

In the present study, we took advantage of this technology to assess the use of feeding sites by roe deer in a peri-urban study area located in the Eastern Italian Alps. Roe deer is a strongly crepuscular [27,28], selective browser [29], with the need to frequently forage due to limited fat reserves [30]. Previous GPS telemetry-based research has shown that in roe deer populations exposed to the availability of supplemental feeding, individuals tended to range in the proximity of feeding sites [14], and to shift their movements in response to the availability of supplemental food [15,16]. However, little is known about roe deer diel and seasonal patterns of feeding site use, and especially on the inter-individual relationship occurring at these concentrated resource sites, which, when rarely observed, evidenced some form of competition and aggressive interactions among individuals [20].

To address this knowledge gap, we firstly analyzed the sex-specific diel patterns of use of these sites within the study population. We hypothesized that the diel pattern in feeding site use would recall the well-known bimodal activity pattern of roe deer (H1; [27]), with characteristic peaks at dawn and dusk for both sexes (P1). Second, we focused on uniquely recognizable individuals (i.e., ear-tagged) to model the frequency of feeding site visits as a function of seasonal trends in temperature and vegetation productivity. We hypothesized that winter and spring visits to feeding sites by roe deer would follow a temporal trend driven by the physiological characteristics of this species (H2). In particular, we expected that the daily number of visits to feeding sites would decrease from winter to spring, concomitant with increasing ambient temperature (P2.1), which we considered as a proxy of the winter harshness in our study system [14]. We also expected that such a pattern would follow the spring trend in vegetation regrowth (P2.2), due to the availability of alternative natural food items on which roe deer could feed. Third, we used the same marked roe deer individuals to model the effect of inter-individual behavior at feeding sites on the duration of the visits. If any form of competition among roe deer occurred for the access to supplemental food, we hypothesized that the duration of the feeding site visits by roe deer would be influenced by the presence of other conspecifics (H3). Specifically, we predicted that, in presence of other individuals, the time spent by the focal roe deer at a given feeding site would increase in response to the trade-off between feeding activity and competition (P3.1). Moreover, we also expected to observe a further increase in visit duration by the focal individual when these co-occurring conspecifics were eating (P3.2). Finally, we assessed whether the diel pattern of use of feeding sites was modulated by inter-individual behavior in the proximity of these concentrated resources, to explore the connections between the physiological and behavioral constraints that drive the use of feeding sites by roe deer. We expected to observe an alteration of the diel pattern of visits to feeding sites in presence of conspecifics, with a shift towards less crowded daily phases (i.e., out of the expected peaks at dawn and dusk), as a tactic to reduce competition when accessing the food at the feeding sites (P3.3).

## 2. Materials and Methods

### 2.1. Study Area

The study area is located in the Italian Eastern Alps (Trento Autonomous Province), in the southern portion of the Cembra Valley. The study area is predominantly hilly and covered by broad-leaved and conifer forest (ca. 80%), and ranges between 600 and 1000 m a.s.l. The climate is relatively continental with sporadic snow cover in wintertime (966 mm annual rainfall; mean daily temperature in January: 1.0 °C; in July: 21.0 °C; source: [31]). Roe deer and red deer (*Cervus elaphus*) are the commonest ungulates in the area (6–9 ind. km^−2^ and 1–2 ind. km^−2^, respectively: reference values from Wildlife Office of the Autonomous Province of Trento [32]), while wild boar (*Sus scrofa*) and chamois (*Rupicapra rupicapra*) presence is sporadic. Other terrestrial mammal species include the European badger (*Meles meles*), European brown hare (*Lepus europaeus*), red squirrel (*Sciurus vulgaris*), and red fox (*Vulpes vulpes*).

The 16 km^2^ study area is covered by a dense network of forestry roads, and two main paved roads that connect five towns (ca. 1000 to 4000 citizens each) located at the borders of the study area, as well as to an urban settlement of regional importance (Trento; 6.5 km in line of sight from the center of the study area). The study area is highly used for recreational activities, and intensively managed for logging and mining. Within the study area, hunters privately manage more than 50 feeding sites (from here below, in Methods and Results: FS (Feeding Site)), which are provided with ad-libitum food (mainly corn and, to a lesser extent, apples) all year-round, as per an ad hoc management plan that had been running for 10 years at the time of the experimental observations (Resolution of the Provincial Government of Trento n. 2852/2013). Selective hunting of roe deer is practiced between September and the end of December from hunting stands, which are most commonly placed within a few hundred meters of the FS.

### 2.2. Monitoring of FS Use by Roe Deer

We monitored a sample of FS by camera traps to identify those mostly used by roe deer and deployed corn-baited wooden box traps for capture at these locations (Supplementary Material 1). Between early December 2016 and early February 2017, we marked seven roe deer with a unique combination of colored and shaped tags on both ears to facilitate their identification. Five individuals (four females and one male) were also fitted with GPS collars (Vectronics Aerospace GmbH), programmed to drop-off after one year, whereas two females were considered too small to be outfitted with the collar. We released the roe deer at their respective capture sites immediately after the conclusion of the handling procedures. We performed captures and marking in compliance with ethical and welfare rules, under the authorization of the Wildlife Committee of the Autonomous Province of Trento (Resolution of the Provincial Government of Trento n. 602, under the approval of the Wildlife Committee of 20 September 2011, and successive integration approved on the 23 April 2015).

At the end of the capture campaign, we started to intensively monitor marked and unmarked roe deer use of FS by camera trapping, focusing on those sites (n = 4) which were being used by the radio-collared roe deer. This led to an overall sampling effort of 769 camera-days between the 20th January 2017 and 31st May 2017 (Supplementary Material 1), when no hunting activity occurred in the area. Each FS was monitored by means of infrared motion-triggered camera traps (models: Stealth Cam G42 and G45, very similar in their technical characteristics and detection capabilities [33]). At each monitored FS, we placed a camera trap within a maximum range of 8 m from the FS, at approximately 70 cm from the ground, and with the focus centered on the FS to maximizing the detectability of roe deer visiting the FS. We programmed the cameras to take photographs with a minimal trigger interval set to 5 s (G42 model) or 30 s (G45 model). We ensured the continuous monitoring of the FS by regular field visits (in full daylight i.e., when roe deer feeding activity is generally low [27]), during which we changed camera trap batteries and we replaced the secure digital (SD) memory card to minimize the risk of data loss due to insufficient data storage. Throughout the monitoring period, we provided FS with ad-libitum food, with the exception of two biweekly periods when food accessibility was restricted (first period: 6th February 2017 to 21st February 2017, regarding two FS; second period: 1st March 2017 to 15th March 2017, regarding a third FS; the fourth FS was always provided with food) for the purpose of an experimental manipulation measuring the responsiveness of roe deer to temporary alteration of food availability (see [15,16] for details).

### 2.3. Processing of Camera Trapping Data

We imported and processed the collected pictures using the Aardwolf 2.0 software [34], which is designed for managing camera trap data in ecological studies. We annotated over 65,000 roe deer pictures collected at the four FS. We recorded ancillary information for each detected individual, including sex, presence of the ear-tag (i.e., individual identification), and behavior at the FS (see Supplementary Material 2 for details). We did not classify individuals by age, considering that the physical appearance on pictures was not sufficient for accurate identification.

We analyzed the diel pattern of FS use by extracting the hourly modes of the timestamp distribution of pictures taken at the FS (i.e., the hour of each day corresponding to the highest number of pictures of a given class), grouped by FS and sex (Supplementary Material 2). We analyzed the seasonal pattern and the influence of conspecifics on the daily number and duration of visits to the FS, respectively, by focusing on the marked roe deer only (N = 6, Table 1; one female was removed since it visited the FS only once). For each combination “FS x marked roe deer”, we defined FS “visits” as the series of pictures detecting the focal marked roe deer, with an interval between two subsequent pictures not exceeding two minutes (i.e., separation time = 2 min). We chose this separation time (i) to catch the determinants of visits to FS at a high spatio-temporal resolution, and (ii) to ensure the consistent identification in successive pictures of the clusters of animals detected within each FS. Indeed, given the difficulty of identifying unmarked roe deer, such consistency would not have been guaranteed with a longer separation time between visits (see Supplementary Material 3 for details and for a sensitivity analysis on the choice of this parameter). We then prepared a dataset containing six time-series (one per individual) of the daily number of visits to FS, and associated covariates: minimum daily temperature; normalized difference vegetation index, NDVI [35]; day of the week; activation status of the FS (see Supplementary Material 2 for details). We also prepared a dataset including all visits from all animals, their duration, and presence of conspecifics (specifically: presence and number of other roe deer, and their feeding activity; see Supplementary Material 2 for details).

### 2.4. Statistical Analyses

#### 2.4.1. Diel Pattern of Feeding Sites Use

For each sex, we plotted rose diagrams, which display the 24 h diel cycle and the frequency of use of each hour, and extracted the circular mean of distributions. In order to assess whether the distributions deviated from uniform, we ran the non-parametric Hermans–Rasson test, which is recommended when circular data follow multimodal distributions [36]. We then performed a pairwise test to compare the male and female distributions. To this end, we firstly carried out the two-sample Rao test [37], to assess the equality of polar vectors and dispersion of the fitted circular data. We then used the two-sample Watson test [38], to compare the homogeneity of the two fitted distributions.

#### 2.4.2. Daily Number of Visits to Feeding Sites

We performed a correlation analysis based on Spearman’s coefficients on a set of biologically relevant candidate covariates to explain the temporal pattern of roe deer visits to FS, finding that the minimum daily temperature, the normalized difference vegetation index (NDVI; [35]), and the day of the year were highly correlated with each other (Supplementary Material 4). We thus ran an analysis of deviance (ANODEV [39]; see Supplementary Material 5 for details) to quantify the percentage of temporal pattern accounted for by the three correlated covariates (P2.1, P2.2). We thus modeled the daily number of visits to FS by means of generalized additive mixed models (GAMMs; [40]; negative binomial distribution of residuals), as a function of the spline of the day of the year, while controlling for the activation status of the FS (i.e., the experimental manipulation, see Supplementary Material 6), and the day of the week (weekday vs. weekend). We fitted a random intercept for individuals to account for potential inter-individual behavioral variation. We applied a model selection procedure based on AIC scores [41] to identify the best model. We then tested the importance of each of the retained terms in reducing the residual deviance of the model by means of an ANOVA based on the Chi-squared test (Supplementary Material 6). Lastly, we measured the percentage of explained deviance computing a pseudo-R squared. We replicated the analysis for different values of the separation time between visits to FS to test the robustness of the model to the choice of this parameter (Supplementary Material 3).

#### 2.4.3. Duration of Visits to Feeding Sites

We firstly assessed, by means of GAMM models [40], whether the number of roe deer observed in a given FS visit decreased significantly over time (Supplementary Material 7) to check for any potential effect of seasonality on the gathering tendency of roe deer. We then modelled the duration of visits to FS by fitting a mixed-effect logistic regression (i.e., a generalized linear mixed model; GLMM) [42]. We firstly checked that the distribution of the response variable i.e., the duration of visits, was non-zero-inflated (Supplementary Material 8). Then, we transformed the duration of visits to FS into a binary response (“short” vs “long” visits), choosing the median of FS visit duration as the cut-off value (see Supplementary Material 9 for a sensitivity analysis on the cut-off choice). We selected a list of biologically relevant candidate covariates that were checked for collinearity (Supplementary Material 4). We then modeled the probability of occurrence of a long or short visit to FS as a function of the presence of other roe deer (binary; present/absent) (P3.1), and their feeding activity (binary; eating/not eating) (P3.2). We fitted these covariates as additional linear effects in the model, together with a random intercept for individuals to account for potential inter-individual behavioral variation. Analogous to what was done for the model predicting the daily number of FS visits, we performed a model selection and simplified the model (Supplementary Material 6), and then tested the sensitivity of our results to the choice of the separation time between visits (Supplementary Material 3). We measured the percentage of deviance explained by the model by computing a pseudo-R squared based on the McFadden formulation [43], and we evaluated the goodness-of-fit of the model by means of an ROC curve and classification table. Lastly, we implemented the circular statistics tests (as in Section 2.4.1) on the circular diel distribution of long and short events (P3.3).

We performed all the statistical analyses, and data preparation using the software R. In particular, we used the packages “chron” [44], “Circular” [45] and “CircStat” [46] for the circular statistics, while “mgcv” [47] and “lme4” [48] were implemented to fit respectively the GAMMs and the GLMMs.

## 3. Results

### 3.1. Diel Pattern of Feeding Sites Use

**Figure 1:**
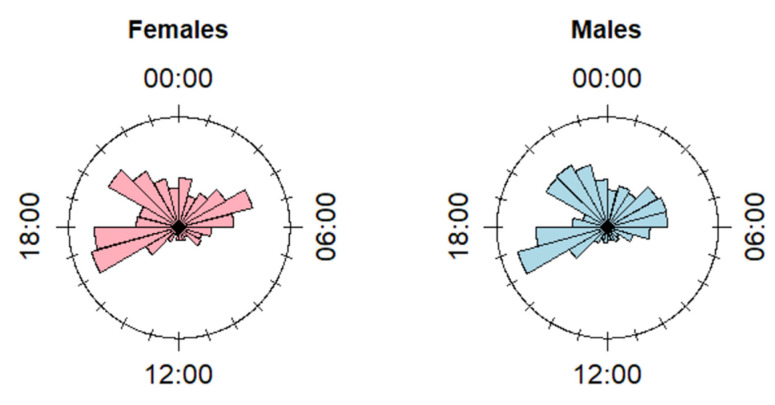
Rose diagrams displaying the circular distribution of the daily modes of the timestamps of the pictures detecting roe deer at FS, divided by sex. Hours shown in UTC.

We performed the diel analysis on a sample of 21 animals, among which 14 females and 7 males (see Supplementary Material 10 for details on the determination of the sample size). Both for females and males, we detected a significantly not homogeneous multimodal distribution of the diel use of FS detected by camera traps (Hermans–Rasson test: *p* < 0.001 for both distributions). In particular, for both sexes, we detected three main peaks of FS use, from 03:00 to 06:00, 16:00 to 18:00, and 20:00 to 23:00 (Figure 1; hours are reported in UTC timezone). Males exhibited a diel pattern of use that was slightly more intense during the night (Figure 1), so that the mean of the resultant vector of the distribution fell at 01:30 (N_daily modes_ = 329), that was significantly later than the mean of the resultant vector for females, which fell at 22:45 (N_daily modes_ = 325) (Rao test: *p* = 0.0018). The Watson test confirmed that the two distributions were not homogeneous (*p* < 0.01).

### 3.2. Daily Number of Visits to Feeding Sites

Over the monitoring period, we obtained 2358 FS visits by the marked roe deer. The daily number of FS visits differed among individuals, ranging between 1 and 672 visits during the total monitoring period (Table 1). With the exception of one roe deer that visited the FS only once, the other six individuals visited the FS 392 ± 230 times during the monitoring period.

**Figure 2:**
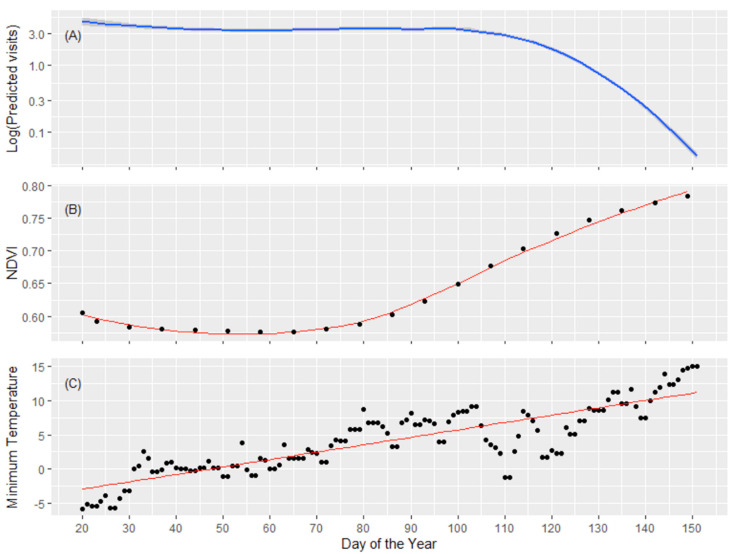
Predictive plot of the daily number of visits to feeding site (log scale) by roe deer as a function of the day of the year (panel **A**) contrasted with the observed increment of NDVI (panel **B**), and minimum daily temperature (panel **C**).

The minimum daily temperature and the normalized difference vegetation index (NDVI) accounted, respectively, for 35.01% and 54.89% of the overall temporal pattern of the daily number of visits to an FS, as shown by the analysis of deviance. The pattern of FS visits changed significantly during the monitoring period (Table 2), with a marked decrease towards the spring, when both NDVI and minimum daily temperature increased (Figure 2) (P2.1, P2.2). The pattern of FS visits varied considerably among individual roe deer (i.e., the random intercept of individuals, Table 2), and increased significantly when the food was accessible at an FS (i.e., activated FS; Table 2). Overall, the model explained 25.80% of the overall deviance (adjusted R-squared = 0.26).

### 3.3. Duration of Visits to Feeding Sites

The mean duration of visits to FS varied among the six marked individuals, ranging from 49 to 382 s, with an average duration of 171 ± 99 s. About a third of the visits (36%) lasted less than 15 s (n = 849). In the majority of the cases the roe deer visited the FS solitarily (n = 1600, 68%). In the remaining cases, one (n = 651, 27%) or more individuals (from two to four; n = 106, 5%) were present contemporarily to the marked focal animal (1.23 to 1.58 individuals on average; Table 1).

**Figure 3:**
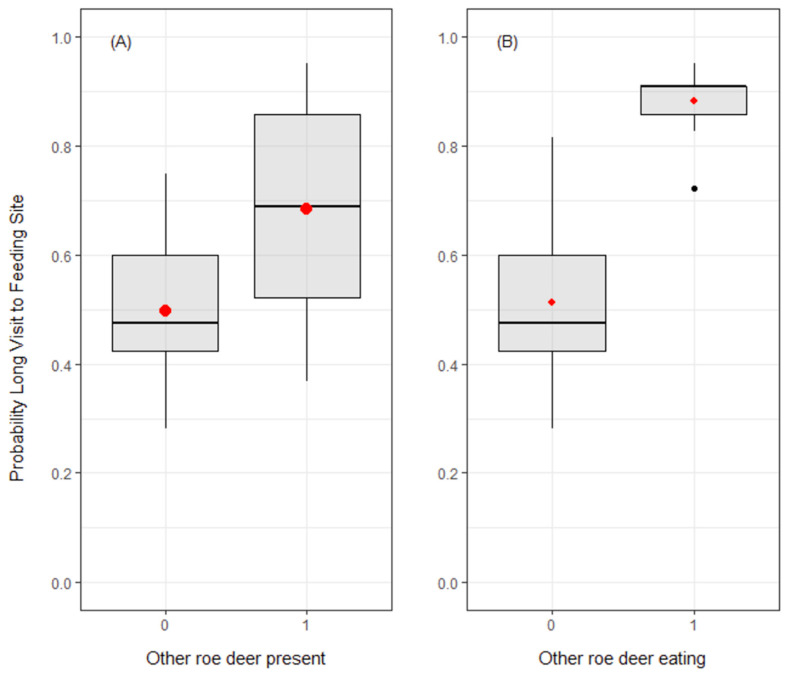
Predictive boxplots reporting the probability to detect a long visit to a feeding site in function of the presence of other roe deer at the feeding site (panel **A**) and of the eating activity of the other roe deer at the feeding site (panel **B**). Red points denote the predicted mean values; the black dot denotes an outlier.

Roe deer probability to gather did not change significantly over the season (Supplementary Material 9). The probability of occurrence of a long visit to FS by a focal roe deer increased in presence of conspecifics at the FS (P3.1: β = 0.39 ± 0.11, *p* < 0.001; reference category: the absence of other roe deer at the FS; Figure 3). Moreover, this pattern was reinforced when the other individual(s) were eating at the FS (P3.2: β = 1.49 ± 0.21, *p* < 0.001; reference category: no eating activity by the other roe deer at the FS; Figure 3). The percentage of deviance explained by the model was equal to 0.08, with 63.70% of the data correctly classified and a ROC area under the curve equal to 0.69 (*p* < 0.001).

**Figure 4:**
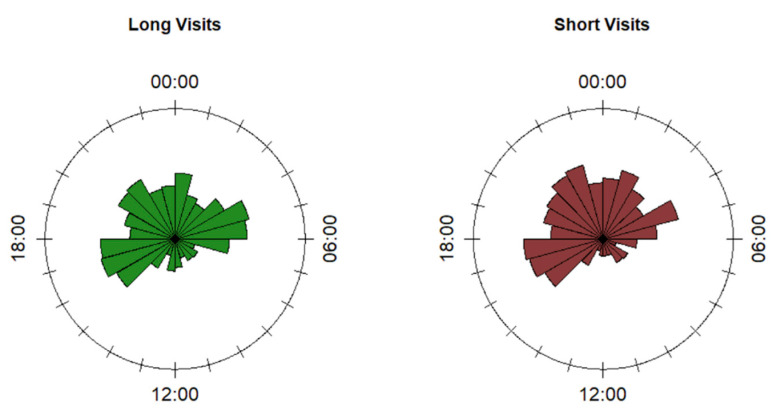
Rose diagrams displaying the circular distribution of the modes of the timestamps of the visits to feeding sites by marked roe deer, grouped by day and feeding site, separated into long and short visits. Hours are shown in UTC.

The diel pattern of FS visits by roe deer did not change in relation to the visit duration (P3.3). For both long and short visits, the distribution of the visits was typically multimodal (Hermans-Rasson test < 0.001), with the main peaks occurring from 04:00 to 06:00, 16:00 to 18:00, and 20:00 to 22:00 (with an extension to 23:00 for short events: Figure 4; hours are reported in UTC timezone). The pairwise tests to compare the two distributions did not show any difference among the means (long visits: 23:10, N_daily modes_ = 243; short visits: 22:55, N_daily modes_ = 211), nor in the homogeneity of the distributions.

## 4. Discussion

The widespread application of ungulate supplemental feeding for a variety of management purposes is surprisingly not supported by in-depth assessments on their patterns of use. By continuously monitoring these concentrated resources with camera traps we provided some of the first empirical evidences that the use of feeding sites by roe deer is opportunistically driven by a combination of physiological, behavioral (‘internal state’, as defined by [49]), and environmental constraints (e.g., winter harshness or resource availability). In particular, we showed that roe deer used these resources following their circadian clock, with the typical bimodal pattern characterized by peaks of activity at dawn and dusk (see e.g., [27,28]). This diel pattern was maintained in the presence of conspecifics, with individuals moderately gathering at feeding sites and staying for a longer time than when visiting them solitarily. Finally, at the seasonal scale, we found that the use of feeding sites by roe deer was inversely related to vegetation productivity and temperature, showing the plasticity of this species to opportunistically switch resources.

The peak of activity during crepuscular hours by roe deer [27,50] has been previously associated with the avoidance of human activities [51]. It is therefore not surprising that such a diel pattern was particularly accentuated for roe deer visits to feeding sites i.e., an anthropogenic setting where individuals are more likely to encounter humans. In our study area, the negative association between feeding sites and humans may also be reinforced by the perceived mortality risk, as several feeding sites are deployed in immediate proximity to hunting stands. Although a proximate mortality risk was not occurring during the monitoring period (i.e., outside of the hunting season), chronic avoidance of these sites in full daylight may have developed (landscape of fear, [52]). Indeed, we speculate that the slightly higher degree of nocturnality observed for males might be linked to particularly severe hunting pressure exerted on males for trophy hunting (see [53] for an example of sex differential movement adaptation in wapiti, and [54] in roe deer in a nearby area to the study one, under the same hunting regime). The sex-biased diel use of feeding sites in relation to hunting risk could be investigated further by comparing sex responses outside and during the hunting season.

Although feeding sites may represent a potential threat, they are a concentrated, highly nutritional food source, particularly attractive for roe deer whose limited rumen capacity leads them to frequently forage on highly energetic items [55], alternating resting and ruminating phases [56]. This alternation emerged in characteristic ultradian activity patterns [27] and habitat use [57,58] and is well reflected in the multi-modal use of feeding sites that we observed for both sexes. After the first peak at dusk, roe deer alternated phases of high and low visit intensity to feeding sites during the night and until early morning (Figure 1). The number of daily visits to the feeding sites by the sub-sample of marked individuals corroborated this pattern: the daily rate of visits to feeding sites (NV/MD, see Table 1) was on average above three visits/night, although with marked inter-individual differences. Interestingly, the alternation of foraging phases at feeding sites could be used as a semi-controlled case to assess periodicity in the use of resources (i.e., recursive behavior [59]) in ungulates, for example using Fourier analysis or spectral representations [60].

The importance of feeding sites as food sources for roe deer is further confirmed by the lack of any modification of the crepuscular activity peaks in the use of these sites when other conspecifics were present. As observed by Bonnot et al. [28], roe deer, as well as other ungulates, are known to maintain the diel activity rhythms, with peaks occurring at crepuscular hours, regardless of potential associated drawbacks. In our context, such drawbacks were associated with the competition to access food at feeding sites, with several individuals gathering there. Our finding that time spent at feeding sites increased in the presence of conspecifics suggests that roe deer tended to tolerate the presence of other individuals, although some anecdotal cases of dominant behavior were also recorded. This observed response could be linked, on the one side, to a low perceived risk, as roe deer rarely exhibit aggressive interactions [20], especially in winter, when small groups may form [61]; on the other, tolerance of conspecifics would be compensated by the advantage of feeding at the times of the day when energetic needs are likely highest, for example at dusk after the low food intake during daylight.

This compensation may not be so advantageous in those systems where predation potentially occurs at feeding sites. In the study area, the stable presence of natural predators has not been observed yet, but the natural and fast recolonization of the grey wolf (*Canis lupus*) [62] will likely impact roe deer diel patterns of use of these concentrated resources in the near future, for example reducing night time spent at feeding sites. The trade-off between predation and anthropogenic non-lethal and lethal disturbance has been shown to shift diel use of habitats [28,63] and activity [50] in roe deer, and feeding site use should make no exception. Similarly, exposure to directly transmitted diseases (e.g., paratuberculosis, [64,65]) increases when contact between individuals become more frequent, and longer [22]. This factor may also decrease the fitness of animals using feeding sites, especially when aggregating. This potential detrimental effect of supplemental feeding merits dedicated research, e.g., through the application of social network analysis [66], aimed in particular to estimate encounter rates between individuals, which is a key parameter in disease transmission models.

In this study, we did not focus on inter-specific relationships at feeding sites, although we anecdotally observed that roe deer and red deer (the other, non-target ungulate intensively using supplemental food) tended to temporally segregate at feeding sites, with the latter being more nocturnal than the former. Other observed animals included badgers, small mammals, and birds. Certainly, analyzing multi-species patterns of use of feeding sites, as proposed by [17,26], would improve our understanding of the ecological role of artificially provided food on animal communities living in managed areas.

Lastly, our results indicate that roe deer tended to modulate their use of feeding sites according to winter severity [14,16] and, especially, in response to the availability of natural food resources. Since roe deer are selective browsers foraging on fresh and highly nutritious vegetation [29], their decreased interest in abundant but repetitive food items provisioned at feeding sites during spring green-up could be expected. In their opportunistic use of the food provided at feeding sites, roe deer confirm their high behavioral and ecological plasticity [67], which is further supported by the observed drastic decrease of visits to feeding sites when supplemental food was not accessible i.e., during the experimental “closure” of feeding sites. The underlying process supporting such behavioral plasticity likely relies on memory, which allows individuals to profitably track spatio-temporal alteration of resource availability [16,68,69]. Interestingly, the seasonal pattern of feeding site use that we describe, and space use and movement behavior around feeding sites [15] varied between roe deer individuals with different degrees of preference for supplemental feeding, supporting the general principle that foraging behavior in mammals may vary widely between individuals [70].

## 5. Conclusions

Our results suggest a tight connection between physiological and behavioral determinants of the use of feeding sites by roe deer. In our system, the tendency to gather in the proximity of these anthropogenic resources was overall limited and, when occurring due to competition for food access, roe deer showed inter-individual tolerance. In turn, the diel foraging rhythms at feeding sites seemed relatively fixed and unaltered by the presence of conspecifics. While being aware that the relatively limited sample size of our case study limits the generalization of our findings, they represent a first, and replicable contribution to unravel the complex relationship between food provisioning and the intra and inter-specific interactions regulating the use of feeding sites. Future studies should evaluate the consequences of such widespread management practice on individual fitness, and population dynamics, with special concern for emergence and transmission of diseases at the species and community level.

## Figures and Tables

**Table 1:** Summary of the visits to feeding sites by individuals. Days of monitoring (DM), number of visits (NV), mean duration (MD), average (ANOD), and maximum number (MNOD) of other roe deer detected during the visits, are shown for each of the seven marked roe deer. Legend: Sex = F (female), M (male). * = animal equipped with GPS collar.

Individual	Sex	DM	NV	MD (Sec)	ANOD	MNOD
1 *	F	132	672	94	1.23	3
2	F	132	219	382	1.59	3
3 *	F	132	628	238	1.36	4
4 *	M	132	485	130	1.41	3
5	F	128	1	210	1	0
6 *	F	126	173	97	1.49	2
7 *	F	114	180	49	1.51	2

**Table 2:** Summary table of the best model for the daily number of visits to feeding sites. For the fixed effects, we reported beta coefficients, and relative standard errors (β ± SE), while for the splines we reported the estimated degrees of freedom (EDF). Legend: Activation (O) = active feeding site, reference category = closed feeding site; S (Day of year) = spline of the day of the year; S (Individual) = spline of the random effect of the individual; ** = 0.001 < *p* < 0.01; *** = *p* < 0.001

	**B ± SE**	**Pr (>|z|)**
Activation (O)	0.82 ± 0.26	0.001 **
	**EDF**	***p*-Value**
S (Day of year)	6.09	<0.001 ***
S (Individual)	4.59	<0.001 ***

## Supplementary Materials

The following are available online at https://www.mdpi.com/2076-2615/10/11/2088/s1, Supplementary Material 1: Description of roe deer captures and monitoring of feeding site use; Supplementary Material 2: Annotation of the pictures and preparation of the datasets for the statistical analyses; Supplementary Material 3: Sensitivity analysis on the separation interval between visits to the feeding sites; Supplementary Material 4: Correlation analysis for the models on daily number and duration of visits to feeding sites; Supplementary Material 5: Analysis of deviance for the model on daily number of visits to feeding sites; Supplementary Material 6: Determination of the best models describing the daily number and duration of visits to feeding sites; Supplementary Material 7: Model describing the pattern of individual gathering tendency throughout seasons; Supplementary Material 8: Assessment of zero inflation pattern in the duration dataset; Supplementary Material 9: Determination of the cut-off threshold to bin the response variable accounting for the duration of visits to the feeding sites; Supplementary Material 10: Assessment of the minimum certain number of roe deer visiting the feeding sites.
