# Supplementary material for: Ecological and Behavioral Drivers of Supplemental Feeding Use by Roe Deer Capreolus capreolus in a Peri-Urban Context
Source: Animals (Basel). 2020 Nov 10;10(11):2088. doi: 10.3390/ani10112088 (PMC7698021; doi:10.3390/ani10112088)
Supplement: Supplementary file 1 [file animals-10-02088-s001.pdf]

SUPPLEMENTARY MATERIAL 1 – Description of roe deer captures and monitoring of feeding site use

We deployed corn-baited box-traps in the immediate surroundings of already existing feeding sites (FS from here below). Specifically, the seven marked roe deer that formed the subsample for the analysis on the daily number and duration of FS visits were captured at four different FS (red and yellow triangles, Figure S1.1). Since two of these were placed at less than 100 m apart from each other, we considered them as a unique capture cluster, defined as 'Site 1' (Figure S1.1). The area comprised between the three capture sites formed a triangle whose size was approximately 1.6 km^2 (Figure S1.1). The capture campaign, which started in early December at all the sites where we attempted to capture roe deer, terminated on January 20th 2017 at Site 1, on January 25th 2017 at Site 2, and on February 6th 2017 at Site 3. We captured four roe deer at the Site 1, two roe deer at the Site 2, and one roe deer at the Site 3 (Table S1.1).

We detail in Table S1.1 the dates of capture, start of monitoring and end of monitoring for each captured roe deer. The monitoring of visits to FS started from the first visit to a given FS by a marked roe deer after the capture event, in order to exclude any bias due to post-release reaction of the individual. The four animals caught at Site 1 were detected either at the same capture site (camera traps CT2 or CT3, see Figure S1.1) or at the FS where we deployed the CT1 (Distance Site 1 – CT 1: 700 m ca. in line of sight). One of the two animals captured at Site 2 (ID = 6) was solely recorded by CT4 (Distance Site 2 – CT4: 580 m ca. in line of sight), while the other roe deer (ID = 5) was detected only once by CT4 and therefore we did not consider it for these analyses. Lastly, the animal caught at Site 3 (ID = 7) was only detected by CT1 (Distance Site 3 – CT 1: 1800 m ca. in line of sight).

Table 1. 1. Site and date of each capture event, and start and end of the monitoring for each marked roe deer.

ID Animal	ID Capture Site	Capture Date	Start Monitoring	End Monitoring
1	Site 1	20.01.2017	20.01.2017	31.05.2017
2	Site 1	02.12.2017	20.01.2017	31.05.2017
3	Site 1	03.12.2017	20.01.2017	31.05.2017
4	Site 1	03.12.2017	20.01.2017	31.05.2017
5	Site 2	23.01.2017	NA	NA
6	Site 2	25.01.2017	26.01.2017	31.05.2017
7	Site 3	06.02.2017	07.02.2017	31.05.2017

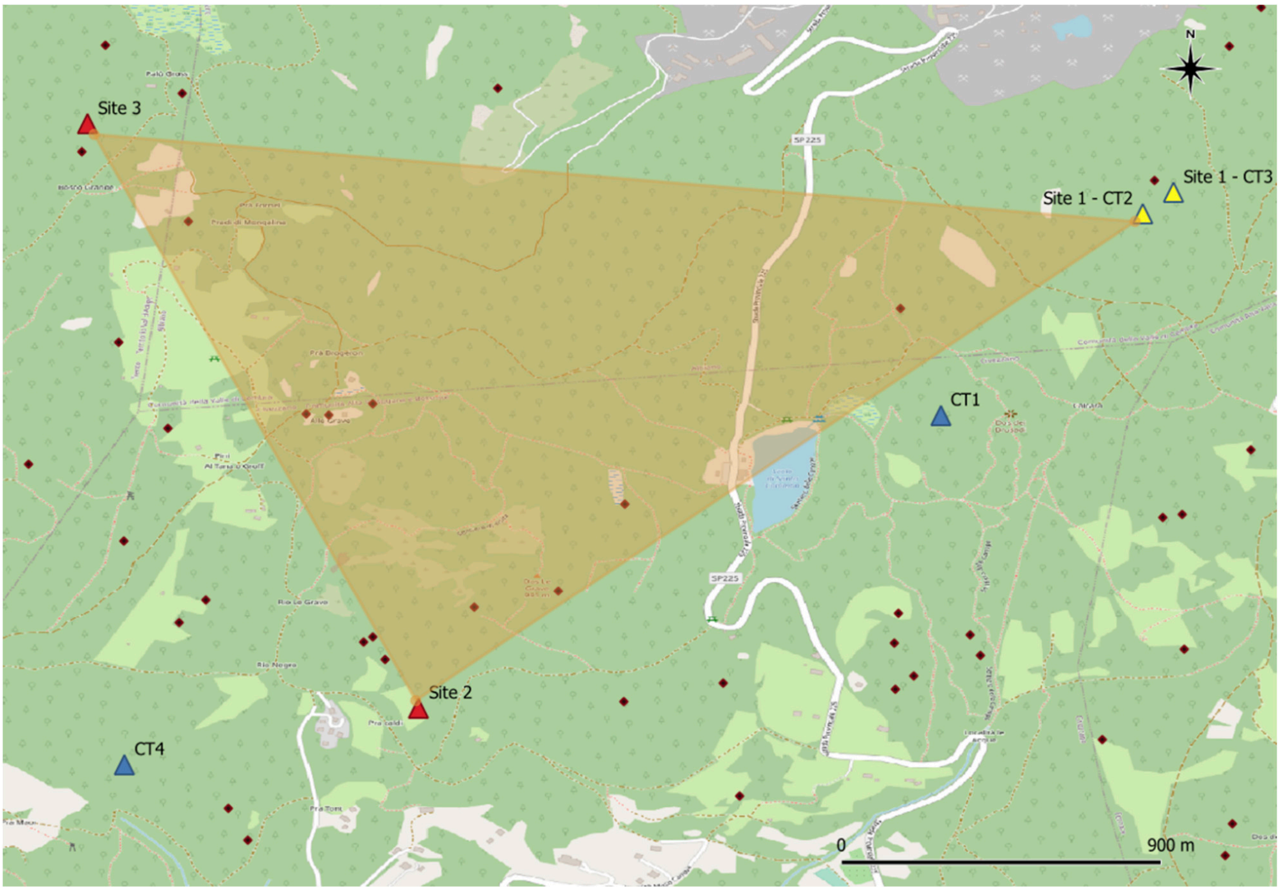

Figure 1. 1. Map of the central part of the study area, where the captures and the monitoring occurred. The red triangles denote the capture sites, the blue triangles represent feeding sites (FS) monitored by means of camera traps (CT), and the yellow triangles denote the two sites that were both used to capture animals and to monitor their use of FS. The orange triangle area has been drawn using the capture sites as vertex. Black small diamonds denote other not monitored FS.

SUPPLEMENTARY MATERIAL 2– Annotation of the pictures and preparation of the datasets for the statistical analyses

Annotation Procedure

Aardwolf is a software specifically designed for managing camera trap data in ecological studies [34] that can manage and organize a large number of pictures. Importantly, Aardwolf enables the user to annotate relevant information (e.g., sex, age class, behavior) to the pictures. Eventually, the managed data can be exported into Comma-Separated Value (CSV) files, where in each row of the dataset the photo ID, the name of the camera, the timestamp of the picture, the Aardwolf metadata and the annotated data are reported.

We used Aardwolf software to annotate the pictures collected by camera traps, and to determine the determinants of the visits to the feeding sites (Fs from here below) by roe deer. The procedure included several steps:

- We determined the beginning of a visit when we could distinguish a roe deer in a given picture. We defined this roe deer as the 'animal_1'.
- For each subsequent picture taken at less than two minutes interval from the previous one (see Supplementary Material 3 for a sensitivity analysis on the separation time between subsequent pictures), we identified any other individual in the visit by a progressive number (hereafter defined as ID) based on the order of appearance in the visit. For instance, the second animal observed in a picture of the same visit was coded as animal_2, the third as animal_3, and so forth. If a given roe deer remained within the field of view for several consecutive pictures, we coded this animal with its first ID code because of the certainty of identification. Instead, when a given roe deer was not detected for one or more pictures of a given visit but reappeared in subsequent pictures of the same visit, we assigned a new ID code to that animal unless we could unequivocally identify it (e.g. based on sex).
- We annotated sex (female, male) of each individual encoded in the visit to the FS, when recognizable. We did not classify individuals by age, because we did not retain the physical appearance on pictures enough informative for an accurate identification.
- We annotated the presence of the ear-tag, so to identify the marked roe deer in a given visit to the FS.
- We also encoded information on the behavior of each roe deer at the FS. These included:
    - **Attendance**: roe deer in proximity of the FS with the capacity of eating without moving its legs, but just moving its neck (Figure S2.1)
    - **Vigilance-attendance**: roe deer in proximity of the FS with (i) the neck turned to see over the feeding site, (ii) the head above shoulder level, (iii) pricked ears, and (iv) roe deer focusing onto one direction (Figure S2.2)
    - **Vigilance in the field of view**: roe deer exhibited a behavior similar to 'vigilance-attendance' but relatively far from the FS (Figure S2.3)
    - **Eating**: roe deer eating at the feeding site, with the head clearly down in the FS and the neck curved (Figure S2.4)
    - **Ground-eating**: roe deer eating outside the FS (supplementary food on the ground, like apples or corn, or natural food in the environment; Figure S2.5)
    - **Ground-attendance**: roe deer exhibiting a behavior similar to the attendance, but meantime eating food on the ground (Figure S2.6)
    - **Feeding from mother**: fawn roe deer feeding from the mother (Figure S2.7)

- **Resting**: roe deer laying on the ground, sleeping or awake (Figure S2.8)
- **Escaping**: roe deer running away (Figure S2.9)
- **Interference-emitter** and **interference-receiver**: two or more roe deer interfering with each other (Figure S2.10). We encoded as interference emitter a roe deer that appeared in the field of view disturbing another already present individual and eventually provoking a reaction of the latter, defined as the interference receiver.

- We encoded information on the presence of other not targeted species in the pictures: badger (*Meles meles*); domestic dog (*Canis lupus familiaris*); cat (*Felis catus*); fox (*Vulpes vulpes*); European brown hare (*Lepus europaeus*); beech marten (*Martes foina*); red deer (*Cervus elaphus*); squirrel (*Sciurus vulgaris*); European jay (*Garrulus glandarius*), wood pigeon (*Columba palumbus*), tawny owl (*Strix aluco*); rodents or birds, when the species was not identifiable.
- We determined the end of a visit when the interval between two subsequent pictures exceeded two minutes.

Dataset for diel pattern of feeding sites use

The preparation of the dataset to assess the diel pattern of use of FS by roe deer included several steps. For this analysis, we relied on 63.110 annotated pictures. We subset the annotated pictures based on sex, thus obtaining two datasets.

Since the pictures are not independent from each other, for each dataset we grouped the pictures on a daily basis and for each FS (i.e. we assumed daily independence of visits to the FS). We then computed, for each day and FS, the mode of the distribution of the picture timestamps, at an hourly resolution. We thus obtained two datasets, each including a time-series of daily modes of visits to FS by females or males. We performed circular statistics analysis on such distributions, as described in the Section 2.3.1 of the main text.

Dataset for daily number of visits to feeding sites

The preparation of the dataset for analyzing the daily number of visits to FS included several steps. For each of the marked roe deer, we firstly prepared a daily time series encompassing the monitoring period, which differed between individuals (see also Supplementary Material 1). Indeed, four individuals had been caught and marked prior to January 20th 2017, and therefore their monitoring period spanned 132 days (from January 20th 2017 to May 31st 2017). For one individual, which was captured on January 25th 2017, the monitoring period spanned 126 days (from January 26th 2017 to May 31st 2017). Lastly, the monitoring of a roe deer captured on February 6th 2017 lasted from February 7th 2017 till May 31st 2017, for a total of 113 days. We then joined with the individual time series the following information:

- The **daily number of visits** to FS, as revealed from the annotation of the mark in the pictures (see above). We fitted a '0' for those days where no visits were recorded.
- The **Minimum Daily Temperature** (source: [31], weather station: Cembra, 690 m a.s.l., ca. 5 km from the study area barycenter in line of sight)
- The **Normalized Difference Vegetation Index** (NDVI; [35]). This is a continuous covariate derived from a MODIS remote sensing index, which is related to the photosynthetic capacity and energy absorption of plant. It provides 8-days resolution time series on the phenological productivity of a given area (spatial resolution: 500 m).
- The **Day of the Week** (0/1), i.e. whether a day is a weekly day or a weekend day.

- The **Activation Status** (0/1) of a given FS, i.e. open or closed for the experiment on roe deer responsiveness to the alteration of food provisioning (see Methods in the main text for a brief description; see also [15-16] for further details).

We thus obtained the datasets for modelling the daily number of visits to the FS in the monitoring period, as described in the Section 2.3.2 of the main text and in Supplementary Material 6.

Dataset for duration of visits to feeding sites

We prepared the dataset for analyzing the duration of visits to FS by marked roe deer by associating to each visit the following information:

- **Duration of the visit** (with start and end timestamps taken from the first and last pictures of the visit). Since we had programmed one out of four camera traps with a coarser trigger delay (30 seconds vs 5 seconds), we standardized the duration of the visits taken by the five-seconds triggers camera traps rounding them to the closest 30′′ resolution (e.g., visits of duration 48′′ were rounded to 60′′, or visits of duration 125′′ were rounded to 120′′). The duration of the visits could vary consistently, from few seconds (e.g. only one picture taken, with at least two minutes gap from the previous and the following one) to several minutes (several pictures in a row with separation time less than two minutes).
- **ID of the FS** where the visit occurred
- **ID** of the focal marked **roe deer**
- **Presence/absence of Other Roe Deer** (0/1)
- **Maximum Number of Roe Deer** observed in a given visit.
- **Behavior of Other Roe Deer**, which we reclassified as 'eating' vs 'not eating' (0/1), thus obtaining the covariate '**Other Roe Deer Eating**'. We assigned the attribute 'eating' if in at least one picture of a given visit another roe deer than the focal one was eating.

We thus obtained the dataset for modelling the duration of visits to FS in the monitoring period, as described in the Section 2.3.3 of the main text and in Supplementary Material 6.

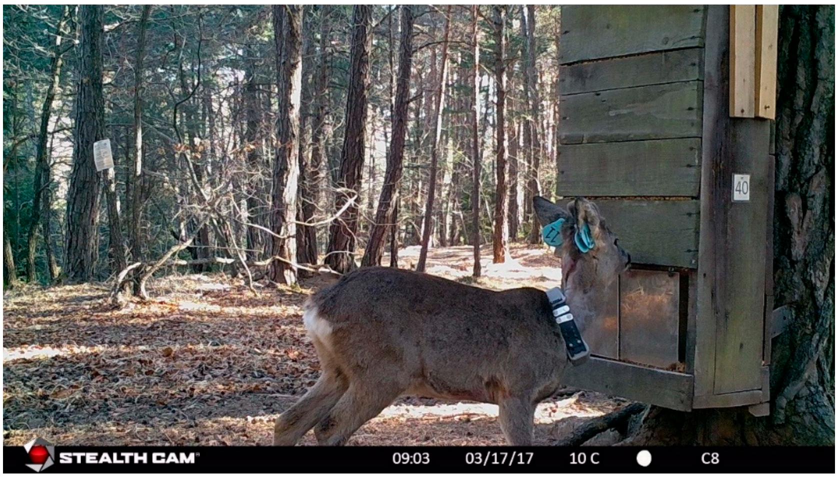

Figure 2. 1. Attendance.

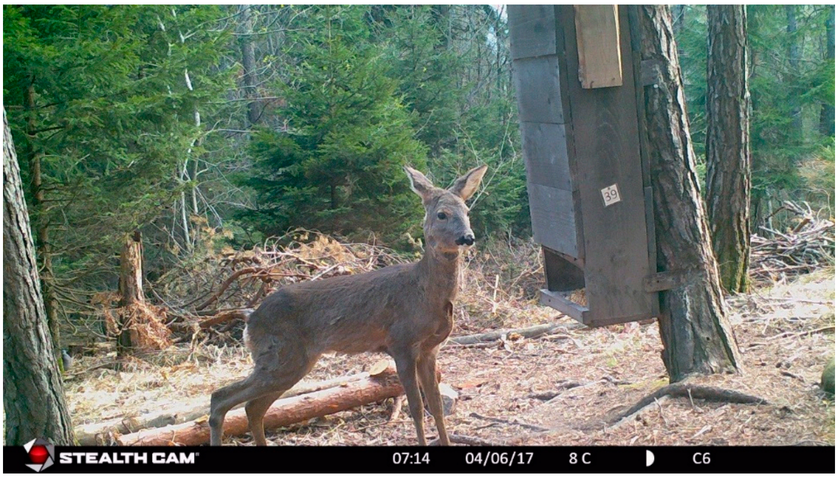

Figure 2. 2. Vigilance-Attendance.

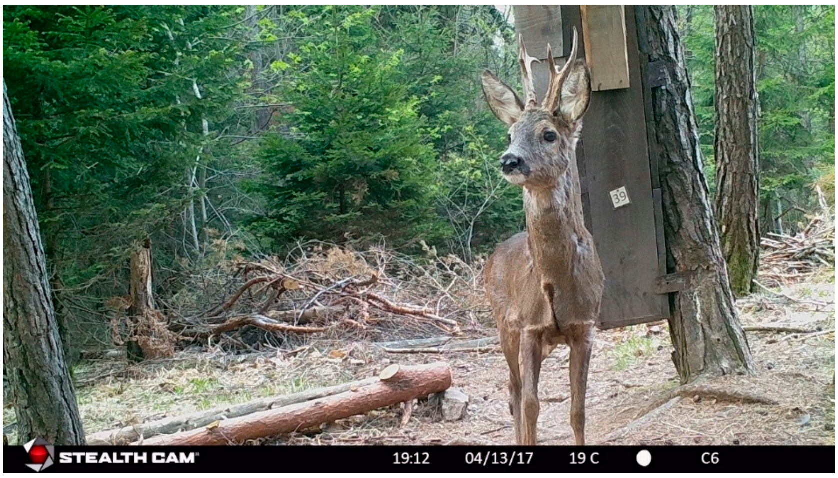

Figure S2.3. Vigilance in the field of view

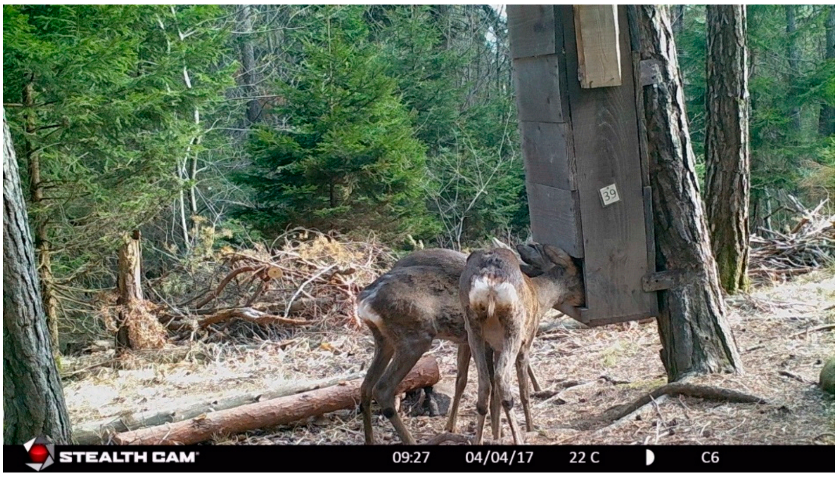

Figure S2.4. Eating

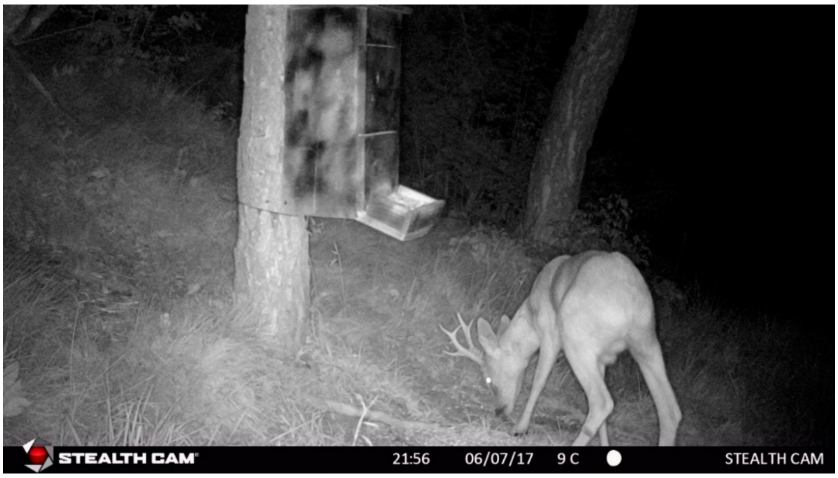

Figure S2.5. Ground eating

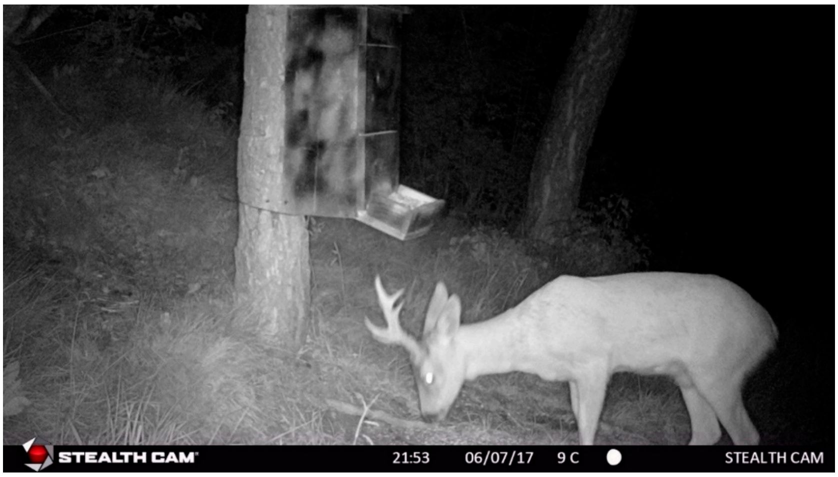

Figure S2.6. Ground attendance

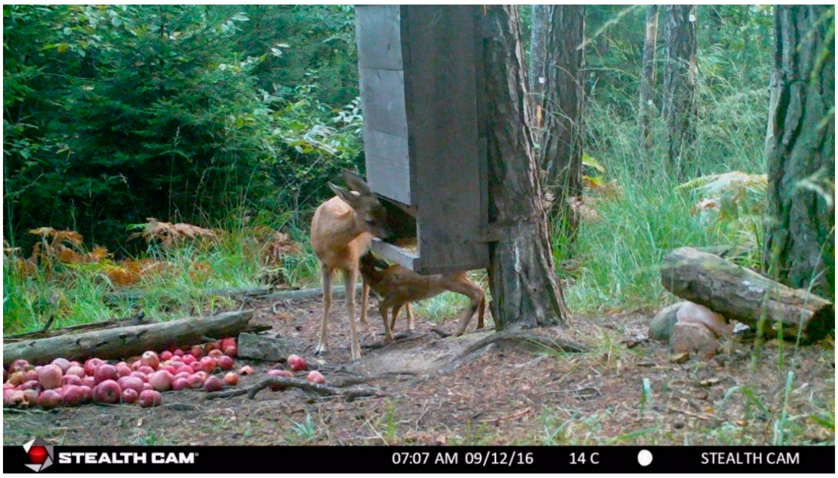

Figure S2.7. Feeding from mother

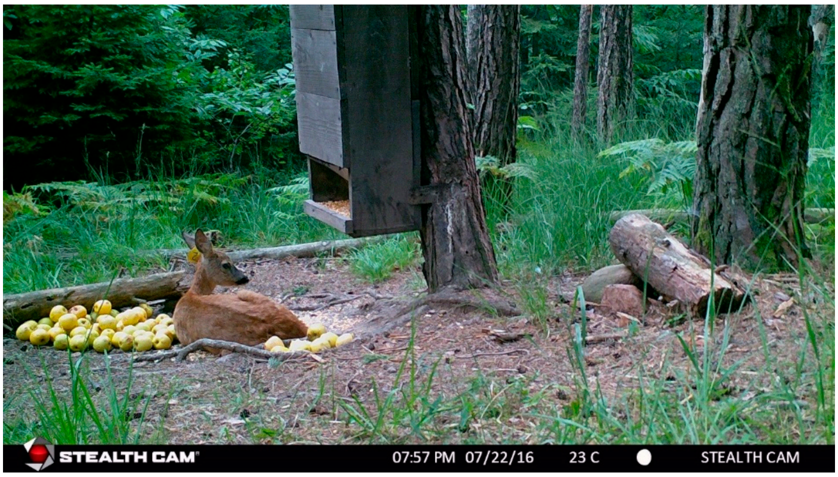

Figure S2.8. Resting

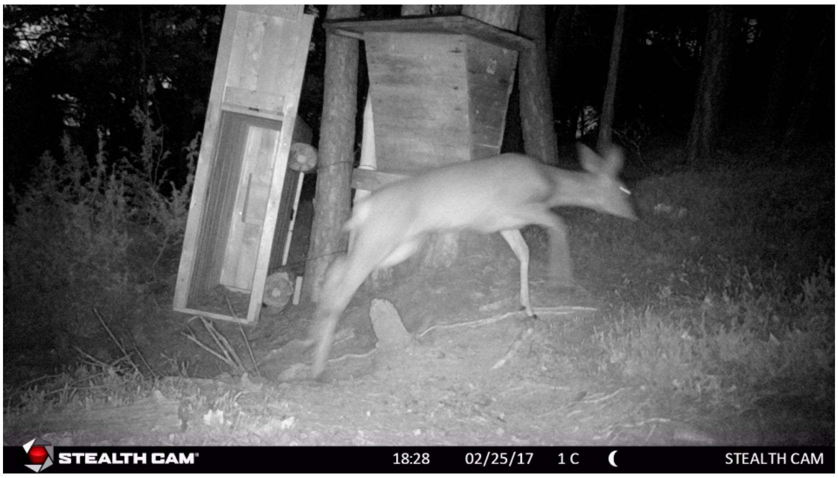

Figure S2.9. Escaping

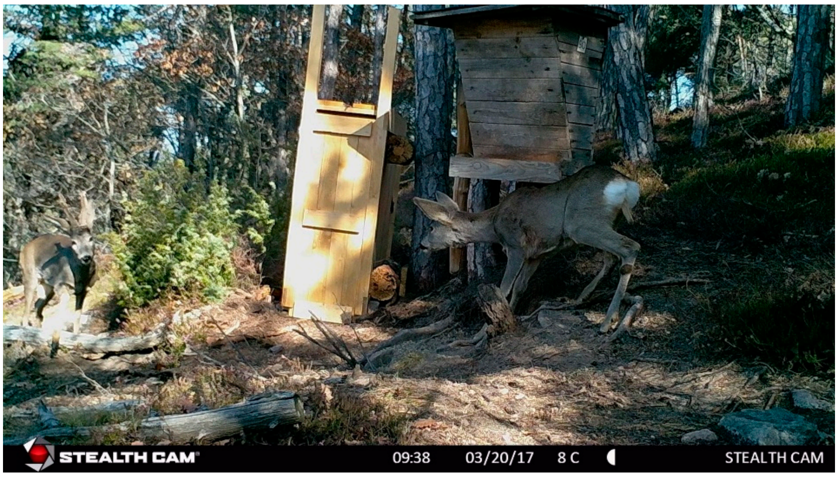

Figure S2.10. Interference

SUPPLEMENTARY MATERIAL 3 – Sensitivity analysis on the separation interval between visits to the feeding sites

The choice of the separation interval that should be set to define independent visits by animals to a given site of interest monitored by camera traps varies with the study context. For instance, Payne et al. [17] opted for 30 minutes to investigate wildlife interactions at baiting sites in an area infected by bovine tuberculosis. In this study, we decided to set the separation interval at a much shorter value, namely two minutes, for two reasons. First, we were interested to catch the determinants of use of feeding sites (from here below: FS) by roe deer at high spatio-temporal resolution, and this could be overlooked when reducing the temporal resolution of the visits to FS (i.e., when increasing the separation interval between subsequent visits). Second, in this work we wanted to assess whether the probability to observe a long visit to a given FS depended on the number of individuals present at the site. Given the difficulty to individually distinguish unmarked roe deer (especially when pictures are taken at night i.e., for the majority of the detections), a higher separation time between visits would have unavoidably hampered our capability to retain within the same visit a stable cluster of individuals. Indeed, with long separation intervals, we cannot assume that unmarked individuals are actually a unique animal, which is instead a reasonable assumption when the separation time between subsequent pictures is short.

We were aware that the choice of this parameter might have influenced the inference about the daily number and duration of visits to FS by roe deer. We thus conducted a sensitivity analysis to assess whether our choice of separation time impacted our findings. Specifically, for both the datasets (daily number and duration of visits), we replicated the statistical analyses with different values of the separation interval i.e., 4, 8, 16, 32 and 64 minutes. The results are reported in Tables S3.1 – S3.3. For what concerns the model describing the duration of visits to FS, we found a significant effect of the factor 'Other roe deer eating', for any tested separation interval (Table S3.1). In addition, the presence of other roe deer at the FS looks overall robust to a change of the separation interval, with two exceptions (no significance for two intermediate separation intervals, i.e. 8 and 16 minutes; Table S3.1). In any case, for none separation interval we detected a change in the sign of the regression coefficient. For each of the tested separation intervals, the individual fitted as random intercept in the model was highly significant (not shown in the table).

With regard to the pattern describing the daily number of visits to FS by roe deer, our results were consistent across the tested separation intervals. Indeed, the fixed effect fitted in the General Additive Model (Activation Status of the FS) was always positively related to the probability of detecting a visit to the FS, with a consistent effect size across the tested separation intervals (Table S3.2). Analogously, the fitted pattern changed significantly over time for each separation interval (Table S3.2), with the percentages of temporal pattern explained by Minimum Daily Temperature and NDVI being highly consistent across all the tested scenarios (Table S3.3). We conclude that our biological findings are not sensitive to the choice of separation interval.

Table 3. 1. Beta coefficients and standard errors for the parameters 'presence of other roe deer ' and 'roe deer eating', describing the probability to detect a long visit to a FS by roe deer, for different separation intervals. Each model also included the random intercept of the individual roe deer (not shown). The stars denote the statistical significance of each covariate in the model. Legend: * = 0.01 < p < 0.05: ** = 0.001 < p < 0.01; *** = p < 0.001.

	**2**	**4**	**8**	**16**	**32**	**64**
Presence	0.39 ± 0.11 ***	0.29 ± 0.13 *	-0.003 ± 0.139	0.09 ±0.15	0.37 ± 0.16 *	0.47 ± 0.16 **
Eating	1.49 ± 0.21 ***	1.33 ± 0.20 ***	1.15 ± 0.19 ***	1.09 ± 0.19 ***	0.47 ± 0.19 *	0.41 ± 0.19 *

Table S3.2. Comparison of the output of the best-supported GAM model describing the daily number of visit to a FS by roe deer, for different separation intervals. For the spline of the day, the

estimated degree of freedoms are reported. The stars denote the statistical significance of each covariate in the model. Legend: * = 0.01 < p < 0.05: ** = 0.001 < p < 0.01; *** = p < 0.001; ACT = Activation Status of the FS; EDF S(Day) = Estimated Degrees of Freedom of the spline of the day.

	**2**	**4**	**8**	**16**	**32**	**64**
ACT	0.82 ± 0.26 **	0.75 ± 0.24 **	0.72 ± 0.23 **	0.71 ±0.22 **	0.73 ± 0.21 ***	0.70 ± 0.20 ***
EDF S(day)	6.098 ***	6.108 ***	6.182 ***	6.270 ***	6.116 ***	6.006 ***

Table S3.3. Comparison of the analysis of deviance procedure (see Supplementary Material 5) that was adopted to compute the percentage of temporal pattern in the daily number of visits to FS by roe deer, explained by the Minimum Daily Temperature (MDT) and by the NDVI, for different separation intervals.

	**2**	**4**	**8**	**16**	**32**	**64**
% MDT	35.01	36.21	36.72	36.86	38.05	39.34
% NDVI	54.89	55.55	54.22	55.22	56.43	56.23

SUPPLEMENTARY MATERIAL 4 – Correlation analysis for the models on daily number and duration of visits to feeding sites

Both for the daily number and duration of visits to feeding sites (FS from here below), we ran pairwise correlation analysis based on Spearman' coefficients to identify potential collinear covariates that could not be fitted within the same model.

Daily number of visits to feeding sites

We found a strong correlation between (i) Day of the Year and the Minimum Daily Temperature (r = 0.84), (ii) NDVI and the Minimum Daily Temperature (r = 0.71) and (iii) Day of the Year and NDVI (r = 0.90). We thus decided to perform an ANODEV procedure [39] to measure the percentage of the overall temporal pattern accounted for by the Minimum Daily Temperature and NDVI (see main text and Supplementary Material 5 for details), while retaining Day of the Year in the model assessing the daily number of visits to FS by roe deer. The other candidate covariates were not highly correlated and therefore we retained them in the models (Table S4.1).

Table 4. 1. Correlation matrix (Spearman' coefficients) among the selected covariates for the analysis of the daily number of visits to FS by roe deer. The relevant correlations are highlighted in bold. Legend: DY= Day of the Year; MDT = Minimum Daily Temperature; NDVI= Normalized Difference Vegetation Index: WD = Weekday or Weekend; ACT = Activation Status of the FS.

	DY	MDT	NDVI	WD	ACT
**DY**	-	**0.84**	**0.90**	-0.01	0.27
**MDT**	-	-	**0.71**	-0.04	0.21
**NDVI**	-	-	-	-0.03	0.24
**WD**	-	-	-	-	0.02
**ACT**	-	-	-	-	-

Duration of visits to feeding sites

We found a strong correlation between the 'Presence of Other Roe Deer' and the 'Maximum Number of Roe Deer' (r =0.93) (Table S4.2). We fitted these two covariates in two different univariate Generalized Linear Models to evaluate their respective power in predicting the duration of visits to FS by roe deer. The model where we fitted the 'Presence of Other Roe Deer' had a lower AIC score (3166.8) than the model including the 'Maximum Number of Roe Deer' at the FS (3179.3). Therefore, we retained for the analysis the covariate 'Presence of Other Roe Deer'. We did not find other relevant correlations, and therefore we retained within the model all the other candidate covariates (Table S4.2).

Table S4.2. Correlation matrix (Spearman' coefficients) of the candidate variables to be fitted in the full model describing the duration of visits to FS by roe deer. The relevant correlations are highlighted in bold. Legend: Presence = Presence of Other Roe Deer; Eating = Other Roe Deer Eating; Max Number = Maximum Number of Roe Deer recorded in the visit to FS.

	Presence	Eating	Max Number
**Presence**	1.00	0.54	**0.93**
**Eating**		1.00	0.55
**Max Number**			1.00

SUPPLEMENTARY MATERIAL 5 – Analysis of deviance for the model on daily number of visits to feeding sites

Since the Minimum Daily Temperature (MDT), the Normalized Difference Vegetation Index (NDVI) and the Day of the Year (DY) were highly correlated (Supplementary Material 4), we could not retain the three of them in the same model predicting the daily number of visits to the feeding sites (FS from here below) by roe deer. As such, we decided to perform an Analysis of Deviance (ANODEV, [39]) to quantify the percentage of temporal pattern explained by MDT and NDVI. To this end, we computed four generalized additive models:

- the simplest null model "S"
- two intermediate models "I" where we predicted the daily number of visits to FS in function of the spline of either the NDVI or MDT
- the most complex model "C" where we predicted the daily number of visits to FS in function of DY as a factor

Both for MDT and for NDVI, we computed the percentage of explained temporal pattern "T" as:

$$T = 1- ((LI-LC))/((LS-LC))$$

where LI = logLikelihood of the intermediate model; LC = logLikelihood of the most complex model; LS = logLikelihood of the simplest model.

SUPPLEMENTARY MATERIAL 6 – Determination of the best models describing the daily number and duration of visits to feeding sites

Both for the daily number and duration of visits to feeding sites (FS from here below), we describe below the procedure that we adopted to determine the best model.

Daily number of visits to feeding sites

The determination of the best model predicting the daily number of visits to FS included different steps. First, we prepared sixteen candidate Generalized Additive Mixed Models, where we fitted the response variable (daily number of visits to FS) in function of all the potential combinations of (i) the spline of the Day of the Year, (ii) the Random Effect of the Individual, (iii) the Activation Status of the FS and (iv) the Day of the Week (weekday/weekend day). The model selection procedure based on AIC scores [41] retained two models (Table S6.1) within a ΔAIC < 2.

Table S6.1. Model selection procedure based on AIC scores. We present the retained models sorted by AIC score (from the lowest to the highest). Legend: s(DY) = spline of the Day of the Year; s(Ind) = random effect of the individual; WD = Weekday or Weekend; AS = Activation Status of the FS.

**Model Structure**	**AIC**
Model 1: Number Visits ~ s(DY) + s(Ind) + AS	3033.6
Model 2: Number Visits ~ s(DY) + s(Ind) + WD + AS	3035.4

We then applied an ANOVA based on deviance procedure to determine the relevance of the 'Day of the Week' that had been retained only in one model (i.e. which had a predictor weight lower than 1; [41]). To do this, we prepared two models, one with the other three covariates (Model1 in Table S6.2), and the other equal to the former with the addition of the covariate 'Day of the Week' (Model2, Table S6.2). The ANOVA based on deviance showed that the covariate 'Day of the Week' did not improve the deviance explained by the model, and therefore we removed it from the model based on the principle of parsimony.

Table S6.2. ANOVA procedure to assess the importance of the Day of the Week in the model. Legend: RD = Residual Deviance, otherwise as for Table S6.1.

**Model Structure**	**RD**	**p-value**
Model 1: Number Visits ~ s(DY) + s(Ind) + AS	3003.8	
Model 2: Number Visits ~ s(DY) + s(Ind) + WD + AS	3003.6	0.676

Lastly, we used the same approach to assess the contribution of each of the three terms with predictor weight equal to 1. To do this, we prepared four models, one containing the three covariates (Model1, Table S6.3) and other three models where we removed each time one of the three covariates (Models 2-4, Table S6.3).

We found that all the covariates significantly increased the deviance explained by the model (Table S6.3), and therefore we retained them in the best model.

Table S6.3. ANOVA procedure to assess the importance of the three covariates with predictor weight equal to 1 in the model. Legend: RD = Residual Deviance, otherwise as for Table S6.1.

**Model Structure**	**RD**	**p-value**
Model 1: Number Visits ~ s(DY) + s(Ind) + AS	3003.8	
Model 2: Number Visits ~ s(DY) + s(Ind)	3013.2	0.002***
Model 3: Number Visits ~ s(DY) + AS	3056.2	<0.001***
Model 4: Number Visits ~ s(Ind) + AS	3141.7	<0.001***

Duration of visits to feeding sites

The determination of the best model accounting for the duration of visits to FS included different steps. First, we prepared a set of candidate Generalized Linear Mixed Models, where we fitted the response variable in function of (i) the Random Effect of the Individual and (ii) each potential additional combination of the two not correlated retained variables (Supplementary Material 4). Following the classical model selection procedure [41], we retained those models with ΔAIC < 2 with respect to the most parsimonious one. The model selection procedure clearly identified a best model, including the covariates 'Presence of Other Roe Deer', 'Other Roe Deer Eating', and the Random Effect of the Individual. We evaluated whether all these covariates significantly increased the deviance explained by the model. To do this, we prepared three models, one including the covariates 'Presence of Other Roe Deer', 'Other Roe Deer Eating', and the Random Effect of the Individual (Model1, Table S6.4), and the other two removing each time one of the three covariates (Models 2-4, Table S6.4). We found that the three covariates significantly increased the deviance explained by the model (Table S6.4). We thus conclude that the best model predicting the occurrence of short or long visits at FS is the one including the covariates 'Presence of Other Roe Deer', 'Other Roe Deer Eating' and the Random Effect of the Individual.

Table S6.4. ANOVA based on deviance procedure to assess the importance of the 'Presence of Other Roe Deer' and 'Other Roe Deer Eating' in the model. Legend: Presence = Presence of Other Roe Deer; Eating: Other Roe Deer Eating; RAN = Random Effect of the Individual; RD = Residual Deviance.

**Model Structure**	**RD**	**p-value**
**Model1**: Duration ~ Presence + Eating + RAN	2968.1	
**Model2**: Duration ~ Eating + RAN	2980.6	<0.001

**Model3**: Duration ~ Presence + RAN	3028.4	<0.001
**Model4**: Duration ~ Presence + Eating	3072.6	<0.001

SUPPLEMENTARY MATERIAL 7 – Model describing the pattern of individual gathering tendency throughout seasons

Roe deer are a generally solitary species, but a tendency to form small groups may be observed in wintertime [60]. Such seasonal effect, if present, may confound the detected trend on roe deer tendency to gather at feeding sites (FS from here below), which we detected (see main text). We thus explored the occurrence of any temporal trend of roe deer tendency to gather at FS along the monitoring period. Specifically, we ran two Generalized Additive Mixed Models (GAMMs; [40]), where we fitted either the Maximum Number of Roe Deer or the Presence of Other Roe Deer as a function of the spline of the Day of the Year. In both models, we fitted individual as random intercept to account for potential inter-individual behavioural variation. Both the models evidenced a significant non-linear temporal pattern, but such pattern did not look to decrease from winter to spring, as one could expect based on the a priori knowledge of the social system of this ungulate. Rather, the predictive plots (Figure S7.1) showed a discontinuous trend in the roe deer tendency to gather at FS, with three peaks along the monitoring period. We conclude that, at least in our study system, roe deer do not evidence a clear seasonal trend from winter to spring in their tendency to form small groups at FS.

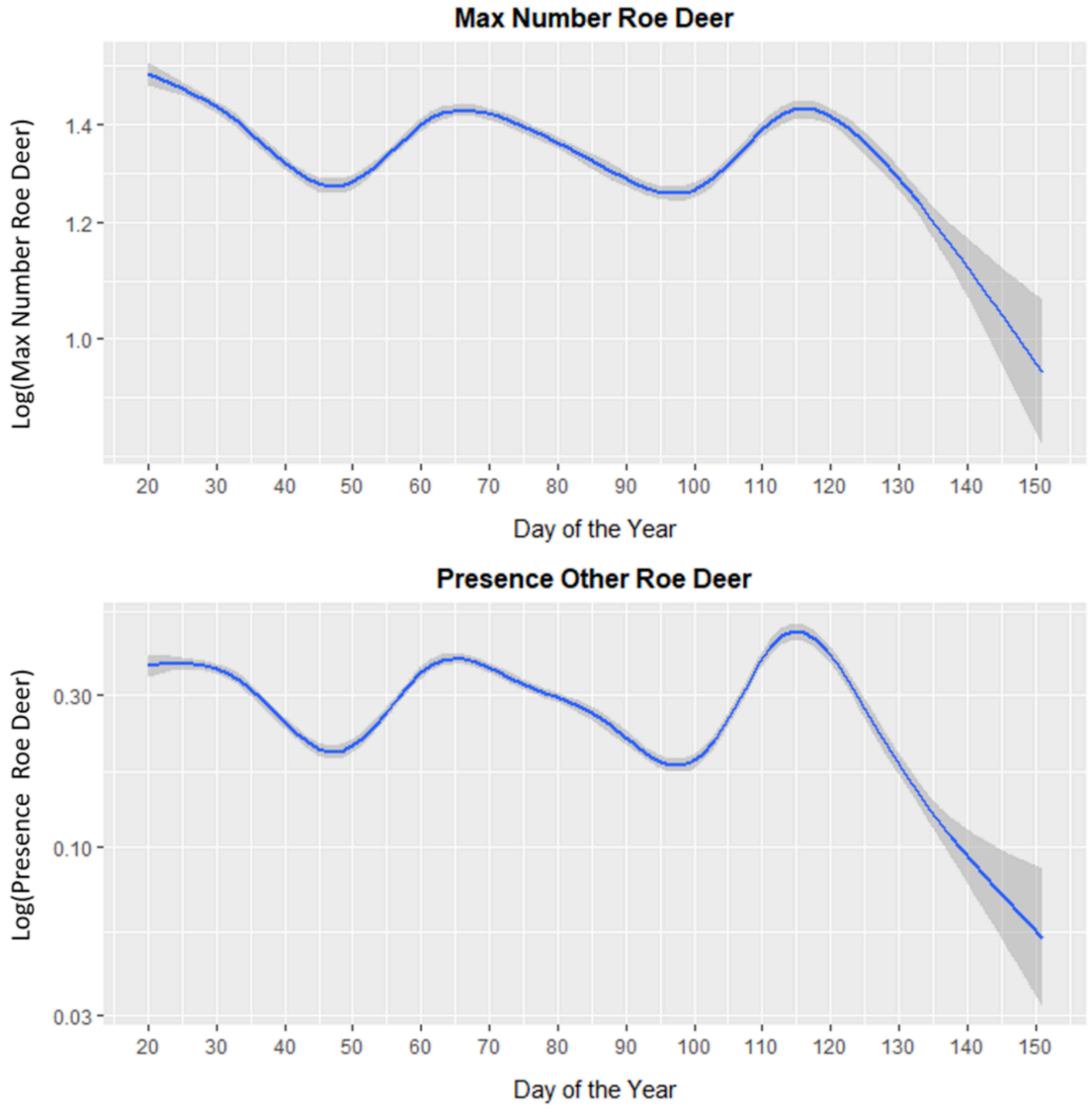

Figure S7.1. Predictive plot of the Maximum Number of Roe Deer (above) and of the Presence of Other Roe Deer (below) in function of the Day of the Year. Shaded areas denote 95% confidence intervals.

SUPPLEMENTARY MATERIAL 8 - Assessment of zero inflation pattern in the duration dataset

The distribution of the duration of visits to the feeding sites (FS from here below) included 849 0s (zeros) occurrences out of 2358 recorded events. We assessed whether the abundance of 0s (zeros) was explained by a particular process with respect to the overall dataset, in which case we should have fitted a zero-inflated distribution. Based on a priori knowledge of the study system, we expected that the excess of 0s (zeros) could be potentially due to the experimental temporary closure of some FS (see main text) and/or to intrinsic behavioral differences among the roe deer. We created two datasets, one including only the 849 visits of duration equal to 0 (zero), and the other with all the recorded visits (n = 2358). Then, both for the FS and for the individuals, we computed the relative proportion of contingencies in each category of the variable, for the 0s and the complete datasets separately (Table S8.1, Table S8.2). For each of the tested variables the proportion of contingencies in each category was overall similar between the 0s (zeros) and the whole dataset. We conclude that there were no reasons to consider the 0s (zeros) as generated by a separate process than the other data, and consequently we did not fit a zero-inflated distribution to the data.

Table 8. 1. Proportion of the contingencies of the visits to FS in function of each of the four monitored FS, for the 0s and the whole dataset.

	**C1**	**C2**	**C3**	**C4**
**0s**	0.47	0.36	0.09	0.08
**All**	0.28	0.39	0.26	0.07

Table 8. 2. Proportion of the contingencies of the visits to FS in function of each individual, for the 0s (zeros) and the whole dataset. Animal 5 was not reported because it was detected only once by camera traps and therefore removed from the analyses (see Table 1 in the main text).

	**1**	**2**	**3**	**4**	**6**	**7**
**0s**	0.36	0.04	0.20	0.21	0.08	0.11
**all**	0.28	0.09	0.26	0.20	0.07	0.08

SUPPLEMENTARY MATERIAL 9 - Determination of the cut-off threshold to bin the response variable accounting for the duration of visits to the feeding sites

The quantile distribution of the rounded duration of visits to the feeding sites (Fs from here below) evidenced that up to 40% of the visits had duration equal to zero (i.e. the visit was made of only one picture, see Supplementary Material 2), while the longest visits lasted almost 2400 seconds (Table S9.1). From the visual inspection of the quantile distribution, it was not obvious to determine a cut-off value as threshold to bin the continuous response variable in short and long visits to FS. We thus performed a sensitivity analysis to evaluate whether the choice of a given cut-off threshold influenced the output of the fitted logistic regression. To do this, we firstly built a full GLMM model where we retained only the not collinear covariates (See Supplementary Material 4 for details) and the random effect of the individual. We fitted as response variable within the model the binned duration of visits to FS, running the model 55 times, once per each quantile from the 40th to the 95th. We excluded a priori from the potential cut-off thresholds those where duration of visits to FS was equal to zero (i.e. from 0th to 39th), as well as the upper 5th percent (i.e. from 95th to 100th). We plotted the beta coefficients and standard errors of each fitted model to evaluate potential discrepancies in the model output due to the choice of the cut-off value (Figure S9.1). For the covariate 'Presence of Other Roe Deer' only slight modifications of the absolute value of the beta coefficient occurred until cut-off thresholds over the 80th quantile, and 'Other Roe Deer Eating' was even more stable for each of the tested cut-offs. We thus decided to use the median of the distribution (i.e. the 50th quantile) as cut-off threshold to bin the duration of visits to FS in short and long visits.

Table S9.1. Quantile distribution of the duration of visits to FS by roe deer.

**Quantile (%)**	**0**	**5**	**10**	**15**	**20**	**25**	**30**	**35**	**40**	**45**	**50**	**55**	**60**	**65**	**70**	**75**	**80**	**85**	**90**	**95**	**100**
**Duration (sec)**	0	0	0	0	0	0	0	0	30	60	60	90	120	120	180	240	300	390	480	660	2370

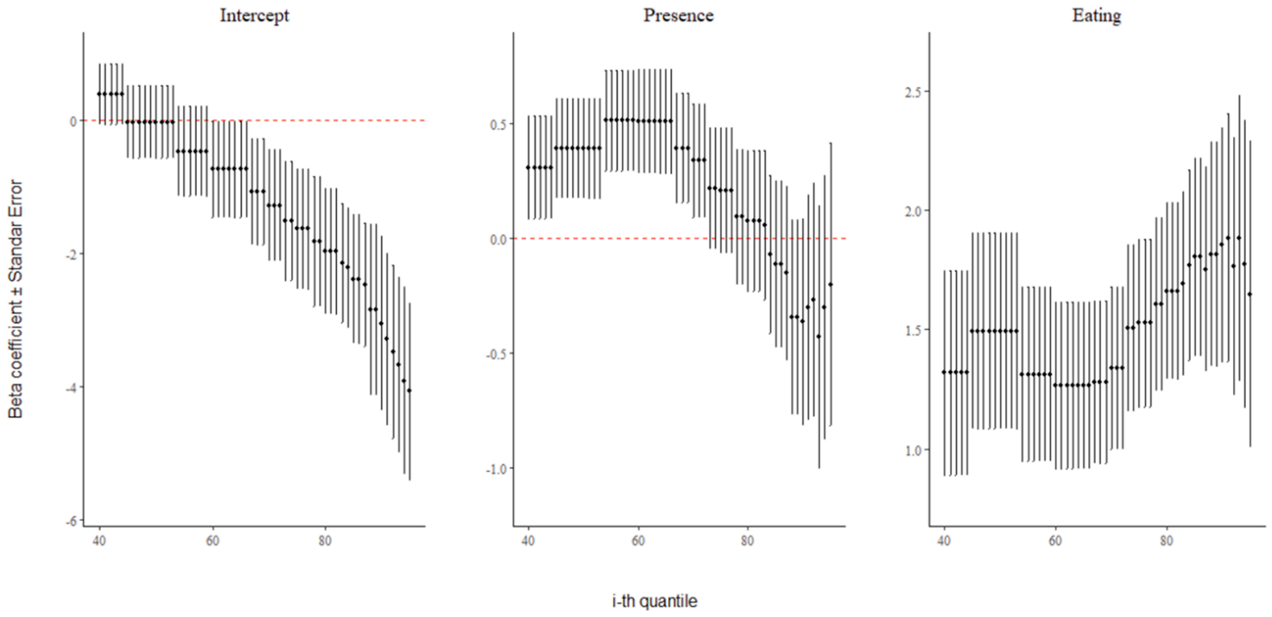

Figure S9.1. Barplot reporting the coefficients (black dots) and related standard errors of each covariate fitted in the model and of the intercept, for each tested quantile (40th – 95th).

SUPPLEMENTARY MATERIAL 10 – Assessment of the minimum certain number of roe deer visiting the feeding sites

We assessed the minimum number of roe deer visiting the feeding sites (FS from here below) adopting a combination of different criteria. First, we considered the monitoring site 'CT4' as separate from the cluster of sites 'CT1, CT2, CT3' (Figure S1.1). We did this because we never observed any marked roe deer detected both at monitoring site CT4 and at one (or more) of the other three monitoring sites. Instead, we frequently observed marked roe deer detected at the monitoring site CT1 visiting also CT2 and CT3 (and vice versa), so that we could not consider these three monitoring sites as independent for this analysis. In other words, we could not be sure that an unmarked roe deer visiting CT1 did not also visit CT2 and/or CT3 along the monitoring period, such risking to double count animals.

For each of the two clusters of monitoring sites (Cluster 1: CT1-CT2-CT3; Cluster 2: CT4), we performed a contingency analysis on the annotated pictures to count the number of females and males detected in each picture. We filtered out from this count the marked animals, in order to count solely the unmarked animals that we observed. We also did not count individuals for which sex was uncertain, because of the risk of double counting individuals. For each of the two clusters, we then added the marked animals observed at those clusters to the counted unmarked individuals. We thus obtained a minimum certain sample of 21 roe deer that we detected by means of camera traps (14 females, 7 males, see Table S10.1). We used all the 21 roe deer for the analysis on diel use of FS based on the sex of the individual.

Table S10.1. Contingency of the animals detected at the clusters of monitoring sites. Legend: F = Female; M = Male; U = Unmarked individual; M = Marked individual. NA = Not Available.

**Monitoring Site**	**FA**	**MA**
CT 1 – CT 2 – CT 3	5U + 4M	3U + 1M
CT 4	3U + 2M	3U
Total	8U + 6M	6U + 1M
